# Supplementary material for: Overlapping cell population expression profiling and regulatory inference in C. elegans
Source: BMC Genomics. 2016 Feb 29;17:159. doi: 10.1186/s12864-016-2482-z (PMC4772325; doi:10.1186/s12864-016-2482-z)
Supplement: Additional file 13: — Web supplement. (DOC 21 kb) [file 12864_2016_2482_MOESM13_ESM.zip › sortWeb/clusters/hier.300.clusters/59.html]

Cluster 59 

## Cluster 59

### Expression

| cnd-1 rep. 1 | cnd-1 rep. 2 | cnd-1 rep. 3 | pha-4 rep. 1 | pha-4 rep. 2 | pha-4 rep. 3 | ceh-27 | ceh-36 | ceh-6 | F21D5.9 | mir-57 | mls-2 | pal-1 | pros-1 | ttx-3 | unc-130 | hlh-16 | irx-1 | ceh-6 (+) hlh-16 (+) | ceh-6 (+) hlh-16 (-) | ceh-6 (-) hlh-16 (+) | cnd-1 singlets | pha-4 singlets | 0 | 60 | 120 | 150 | 180 | 240 | 330 | 390 | 420 | 480 | 540 | 570 | 600 | 630 | 660 | NAME | Functional description |
| --- | --- | --- | --- | --- | --- | --- | --- | --- | --- | --- | --- | --- | --- | --- | --- | --- | --- | --- | --- | --- | --- | --- | --- | --- | --- | --- | --- | --- | --- | --- | --- | --- | --- | --- | --- | --- | --- | --- | --- |
|  |  |  |  |  |  |  |  |  |  |  |  |  |  |  |  |  |  |  |  |  |  |  |  |  |  |  |  |  |  |  |  |  |  |  |  |  |  | *linc-120* | Long Intervening Non-Coding RNA |
|  |  |  |  |  |  |  |  |  |  |  |  |  |  |  |  |  |  |  |  |  |  |  |  |  |  |  |  |  |  |  |  |  |  |  |  |  |  | C54H2.6 |  |
|  |  |  |  |  |  |  |  |  |  |  |  |  |  |  |  |  |  |  |  |  |  |  |  |  |  |  |  |  |  |  |  |  |  |  |  |  |  | K09E3.4 |  |
|  |  |  |  |  |  |  |  |  |  |  |  |  |  |  |  |  |  |  |  |  |  |  |  |  |  |  |  |  |  |  |  |  |  |  |  |  |  | C04D8.3 |  |
|  |  |  |  |  |  |  |  |  |  |  |  |  |  |  |  |  |  |  |  |  |  |  |  |  |  |  |  |  |  |  |  |  |  |  |  |  |  | F26A3.5 |  |
|  |  |  |  |  |  |  |  |  |  |  |  |  |  |  |  |  |  |  |  |  |  |  |  |  |  |  |  |  |  |  |  |  |  |  |  |  |  | C36F7.18 |  |
|  |  |  |  |  |  |  |  |  |  |  |  |  |  |  |  |  |  |  |  |  |  |  |  |  |  |  |  |  |  |  |  |  |  |  |  |  |  | Y41C4A.6 |  |
|  |  |  |  |  |  |  |  |  |  |  |  |  |  |  |  |  |  |  |  |  |  |  |  |  |  |  |  |  |  |  |  |  |  |  |  |  |  | F28D1.6 |  |
|  |  |  |  |  |  |  |  |  |  |  |  |  |  |  |  |  |  |  |  |  |  |  |  |  |  |  |  |  |  |  |  |  |  |  |  |  |  | R08B4.5 |  |
|  |  |  |  |  |  |  |  |  |  |  |  |  |  |  |  |  |  |  |  |  |  |  |  |  |  |  |  |  |  |  |  |  |  |  |  |  |  | Y116A8C.40 |  |
|  |  |  |  |  |  |  |  |  |  |  |  |  |  |  |  |  |  |  |  |  |  |  |  |  |  |  |  |  |  |  |  |  |  |  |  |  |  | *odr-7* | ODoRant response abnormal |
|  |  |  |  |  |  |  |  |  |  |  |  |  |  |  |  |  |  |  |  |  |  |  |  |  |  |  |  |  |  |  |  |  |  |  |  |  |  | *tag-4* | Temporarily Assigned Gene name |
|  |  |  |  |  |  |  |  |  |  |  |  |  |  |  |  |  |  |  |  |  |  |  |  |  |  |  |  |  |  |  |  |  |  |  |  |  |  | *acbp-7* | Acyl-Coenzyme A Binding Protein |
|  |  |  |  |  |  |  |  |  |  |  |  |  |  |  |  |  |  |  |  |  |  |  |  |  |  |  |  |  |  |  |  |  |  |  |  |  |  | C54D2.10 |  |
|  |  |  |  |  |  |  |  |  |  |  |  |  |  |  |  |  |  |  |  |  |  |  |  |  |  |  |  |  |  |  |  |  |  |  |  |  |  | *anr-20* | Antisense Non-coding RNA |
|  |  |  |  |  |  |  |  |  |  |  |  |  |  |  |  |  |  |  |  |  |  |  |  |  |  |  |  |  |  |  |  |  |  |  |  |  |  | T12A7.11 |  |

### Phenotypes enriched

none found

### Anatomy terms enriched

none found

### GO terms enriched

none found

### Expression clusters enriched

none found

### Motifs enriched

|  |  |  |  |  |  |
| --- | --- | --- | --- | --- | --- |
| **Motif** | **Logo** | **Possible orthologs** | **Number of motifs in cluster** | **Enrichment** | **FDR corrected p** |
| Pax6\_3838 |  | alr-1 (0.6) lim-6 lim-7 lim-4 dsc-1 | 16 | 1.87 | 0.0055 |
| pTH9052 |  | ces-2 | 12 | 2.63 | 0.0140 |
| ESRRA\_3 |  | nhr-71 | 9 | 3.58 | 0.0200 |
| Cart1\_0997 |  | alr-1 (0.6) | 14 | 1.90 | 0.0480 |

### Correlated (and anti-correlated) transcription factors

|  |  |
| --- | --- |
| **Transcription factor** | **Correlation** |
| odr-7 | 0.87 |
| fkh-5 | 0.73 |
| hlh-19 | 0.69 |
| unc-86 | 0.64 |
| ZK337.2 | 0.62 |
| nhr-187 | 0.61 |
| alr-1 | 0.60 |
| nhr-67 | 0.58 |
| nhr-216 | 0.58 |
| ceh-30 | 0.57 |
| nhr-188 | 0.56 |
| ets-5 | 0.56 |
| ceh-90 | 0.56 |
| tab-1 | 0.56 |
| hlh-4 | 0.53 |
| elt-6 | 0.52 |
| F47E1.3 | 0.52 |
| T23F11.4 | 0.49 |
| nhr-94 | 0.47 |
| lag-1 | 0.47 |
| hlh-12 | 0.46 |
| ceh-8 | 0.46 |
| lin-11 | 0.45 |
| egl-13 | 0.45 |
| nhr-55 | 0.44 |
| ubxn-1 | -0.39 |
| lin-22 | -0.39 |
| nhr-2 | -0.40 |
| aptf-2 | -0.41 |
| pie-1 | -0.41 |
| hmbx-1 | -0.41 |
| nhr-108 | -0.42 |
| nhr-176 | -0.43 |
| T26A5.8 | -0.44 |
| nhr-169 | -0.45 |
| F49E8.2 | -0.48 |
| pax-3 | -0.49 |
| nhr-257 | -0.49 |
| nhr-271 | -0.49 |
| mex-5 | -0.49 |
| F17C11.1 | -0.50 |
| ceh-81 | -0.50 |
| xnd-1 | -0.50 |
| ztf-28 | -0.53 |
| pqn-75 | -0.55 |
| nhr-210 | -0.55 |
| pzf-1 | -0.55 |
| cey-2 | -0.60 |
| nhr-248 | -0.63 |
| nhr-171 | -0.64 |

### ChIP peaks enriched

none found
